# Doxorubicin treatment induces tumor cell death followed by immunomodulation in a murine neuroblastoma model

**DOI:** 10.3892/etm.2014.1489

**Published:** 2014-01-17

**Authors:** SEIICHIRO INOUE, YUMIKO SETOYAMA, AKIO ODAKA

**Affiliations:** 1 Department of Hepato-Biliary-Pancreatic and Pediatric Surgery, Saitama Medical Center, Saitama Medical University, Kawagoe, Saitama 3508550, Japan; 2 Department of Medical Research, Saitama Medical Center, Saitama Medical University, Kawagoe, Saitama 3508550, Japan

**Keywords:** immunogenic cell death, neuroblastoma, innate cellular immunity, doxorubicin, cisplatin

## Abstract

Chemotherapy of malignant tumors induces tumor cell death. Numerous antitumor agents induce apoptosis of tumor cells, which are subsequently engulfed by phagocytes, initiating an immune reaction. The induction of immunogenic cell death by antitumor agents may be advantageous for antitumor immunity. The purpose of this study was to determine whether doxorubicin is capable of inducing an immunogenic reaction in murine neuroblastoma cells. The murine neuroblastoma cell line (neuro-2a cells) was cultured in a medium containing doxorubicin or cisplatin (CDDP), and induction of cell death was confirmed by cell viability assays. Cluster of differentiation (CD)8α^+^ lymphocytes were co-cultured with neuro-2a cells that had died following treatment with either doxorubicin or CDDP, and CD11b^+^ spleen cells or bone marrow-derived dendritic cells (BM-DCs) were added to the culture. Proliferation of CD8α^+^ lymphocytes and interferon (IFN)-γ production were evaluated. When CD8α^+^ cells were co-cultured with doxorubicin-treated neuro-2a cells and BM-DCs, CD8α^+^ cells reacted to anti-CD3/CD28 antibody stimulation, proliferated and increased IFN-γ production. IFN-γ production was more effectively promoted by co-culture with doxorubicin-treated neuro-2a cells than by co-culture with CDDP-treated neuro-2a cells. These findings suggest that doxorubicin is capable of inducing immunogenic cell death in neuroblastoma cells, and thus has an immunological advantage for chemotherapy of neuroblastoma compared with CDDP. BM-DCs are considered to be the key antigen-presenting cells in the immune reaction following the induction of immunogenic neuroblastoma cell death and phagocytosis.

## Introduction

Chemotherapy combined with radiation and surgery is an important component of intensive therapies for high-risk neuroblastoma. Combination therapy with multiple antitumor agents induces tumor cell death. The primary mechanisms of cell death are apoptosis, necrosis and autophagy. In particular, apoptosis and necrosis have significantly different effects on the subsequent immunological response (1,2). Apoptotic cells are commonly engulfed by phagocytes, such as macrophages or dendritic cells (DCs), which induce a sequential immune response (1,2). Whether the engulfed apoptotic cells induce subsequent tolerogenicity or immunogenicity depends on the function of the phagocytes involved. Furthermore, it has been revealed that the mechanisms of cell death induced by chemotherapeutic antitumor agents have an effect on the immunogenicity of dying tumor cells (2–4). Obeid *et al* (5) and Martins *et al* (6) reported that anthracyclins induce immunogenic tumor cell death in a murine colon cancer model. In this model, anthracyclins induced the translocation of calreticulin (CRT) to the cell surface, and CRT was exposed by dying tumor cells phagocytosed by DCs, resulting in the presentation of tumor antigens and the induction of immunogenicity in tumor cells (5,6). This evidence has provided insights into the immunological benefits and drawbacks of conventional chemotherapeutic antitumor agents. Moreover, a clear understanding of the cellular basis of immunogenicity induced by dead tumor cells treated with chemotherapeutic agents is likely to provide novel strategies for the development of therapeutic vaccines for advanced cancer (4,7). To date, only a few studies have investigated the immunogenic effect of anthracyclins on neuroblastoma cells (8).

Despite the availability of intensive, multimodal treatments, the long-term survival of patients with high-risk neuroblastoma remains unsatisfactory (9–11). The majority of high-risk neuroblastomas respond to initial therapy but subsequently relapse, and it has been suggested that tumor cells may acquire drug resistance following multi-agent chemotherapy. However, a passive, antibody-based immunotherapeutic approach increased the two-year event-free survival rate, indicating that immunological mechanisms are capable of promoting the eradication of high-risk neuroblastoma cells (10,12). Investigation into the potential immunological benefits of chemotherapeutic agents may improve conventional chemotherapeutic regimens and help to establish novel immunological therapies for high-risk neuroblastoma (13).

In this study, doxorubicin was administered to a murine neuroblastoma cell line *ex vivo* to induce tumor cell death, and the immunogenicity of the dead tumor cells was examined. In addition, the mechanism underlying the immune reaction following phagocytosis of the dead neuroblastoma cells was investigated.

## Materials and methods

### Murine tumor cell line

The murine neuro-2a neuroblastoma cell line (H2-K^a^, CCL-131), which was developed in A/J mice, was purchased from the American Type Culture Collection (ATCC; Manassas, VA, USA). Cells were maintained in Minimum Essential Medium (MEM) with 10% fetal bovine serum (ATCC) and 1% penicillin-streptomycin (10,000 U/ml; Gibco; Invitrogen Life Technologies, Carlsbad, CA, USA) at 37°C in 5% CO_2_.

### Animals

Female A/J mice (H2-K^a^; Japan SLC, Inc., Hamamatsu, Japan), aged 6–10 weeks, were maintained under standard conditions. The Ethics Committee of The animal experiment, Saitama Medical University, Saitama, Japan, approved the animal procedures.

### Induction of tumor cell death and cell survival assay

Cell death induced by doxorubicin (Sigma-Aldrich, St. Louis, MO, USA) and cisplatin (CDDP; Maruko^®^, Yakult, Tokyo, Japan) was examined using the cell viability reagent water-soluble tetrazolium salt 8 (WST-8; Wako Chemicals, Osaka, Japan), according to the manufacturer’s instructions. Briefly, neuro-2a cells (0.40×10^5^ cells/90 μl/well) were plated in 96-well plates and incubated overnight. Subsequently, 10 μl MEM with 10% fetal bobine serum and 1% penicillin-streptmycin combined with either doxorubicin (final concentration, 6.1×10^−3^-100 μM) or CDDP (final concentration, 1.0×10^−7^-1.0×10^−1^ mg/ml) was added to each well, and the mixture was incubated for either 24 h (doxorubicin) or 72 h (CDDP). WST-8 (10 μl/well) was added, and, after 2 h incubation, absorbance at 450 nm was measured with a microplate reader (Thermo Fisher Scientific, Yokohama, Japan). Experiments were repeated at least three times to confirm that the results were reproducible.

### Isolation of cluster of differentiation (CD)11b^+^ spleen cells from A/J mice

CD11b^+^ spleen cells were prepared as antigen-presenting cells (APCs). Whole spleen cell suspensions were prepared by passing spleen tissue minced with scissors through a 70-μm cell strainer (BD Biosciences, San Diego, CA, USA). Erythrocytes were lysed in an erythrocyte lysis solution (BD Pharm Lyse™; BD Biosciences) and washed with RPMI-1640 medium containing 10% fetal calf serum (FCS). The cells were then re-suspended in MACS^®^ running buffer (Miltenyi Biotec GmbH, Bergisch Gladbach, Germany), incubated with CD11b^+^ magnetic beads (Miltenyi Biotec GmbH) for 15 min at 4°C, and positively sorted using an autoMACS^®^ Pro Separator (Miltenyi Biotec GmbH). CD11b^+^ cells were re-suspended in RPMI-1640 medium.

### Generation of bone marrow-derived DCs (BM-DCs)

BM cells were harvested from A/J mice by flushing the femurs and tibias with RPMI-1640 medium (Gibco; Invitrogen Life Technologies). The BM material was passed through a 70-μm cell strainer (BD Biosciences) and the cells were centrifuged (440 × g at room temperature) and re-suspended in erythrocyte lysis solution. The surviving cells were washed with RPMI-1640 medium containing 10% FCS, prior to being re-suspended in RPMI-1640 medium supplemented with 10% FCS, 50 μM 2-ME (Sigma-Aldrich), 1% MEM non-essential amino acid solution and 1% penicillin-streptmycin solution (Gibco; Invitrogen Life Technologies). Following the addition of recombinant mouse granulocyte-macrophage colony-stimulating factor (GM-CSF; 20 ng/ml; R&D Systems, Inc., Minneapolis, MN, USA), the cells were plated and incubated at 37°C in 5% CO_2_. Fresh medium containing 20 ng/ml GM-CSF was added after three days of incubation, and adherent cells were harvested by trypsinization after seven days. The expression of CD11c and major histocompatibility complex (MHC) class II antigens was assessed by fluorescence-activated cell sorting (FACS) using phycoerythrin (PE)-conjugated anti-mouse CD11c antibodies (Invitrogen Life Technologies) and fluorescein isothiocyanate-conjugated anti-MHC class II antibodies (Miltenyi Biotec GmbH).

### In vitro CD8α^+^ T-cell proliferation assay and interferon (IFN)-γ detection by ELISA

To evaluate the immunogenicity of doxorubicin-treated, dead neuro-2a cells, doxorubicin-treated neuro-2a cells were co-cultured with CD8α^+^ cells and proliferation of the CD8α^+^ cells was evaluated. The inguinal and mesenteric lymph nodes (LNs) and spleens were removed from A/J mice. The LNs were ground between two glass slides, washed with RPMI-1640, passed through a 70-μm cell strainer and re-suspended in MACS running buffer. The cells were then incubated with CD8α magnetic beads (Miltenyi Biotec GmbH) for 15 min at 4°C, and positively sorted using the autoMACS^®^ Pro Separator (Miltenyi Biotec GmbH). CD8α^+^ cells were labeled with 10 μM carboxyfluorescein succinimidyl ester (CFSE; Enzo Life Sciences Inc., Plymouth Meeting, PA, USA) and re-suspended in RPMI-1640 supplemented with 10% FCS, 50 μM 2-ME, 1% MEM-non-essential amino acid solution, 1% penicillin-streptmycin and MEN vitamin solution (Gibco; Invitrogen Life Technologies). Neuro-2a cells (2.0×10^5^) that had been treated with doxorubicin (final concentration, 5 μM) for 24 h, as well as CFSE-labeled CD8α^+^ cells (2.0×10^5^) and CD11b^+^ spleen cells (0.5×10^5^), were plated in 24-well flat-bottomed plates coated with hamster anti-mouse CD3/CD28 antibodies (BD Pharmingen, San Diego, CA, USA) in 1 ml RPMI supplemented with 10% FCS, 50 μM 2-ME, 1% MEM-non-essential amino acid solution, 1% penicillin-streptmycin and MEN vitamin solution. As an adjuvant, purified, single-stranded CpG-oligodeoxynucleotide (ODN)-1826 (5′-TCCATGACGTTCCTGACGTT; Nippon Gene Co., Ltd., Toyama, Japan) was added to each well (10 μg/well). The cells were incubated for three days at 37°C in 5% CO_2_, and then harvested. CFSE dilution of the CD8α^+^ cells was evaluated using the FACScan system (BD Biosciences). Concentrations of IFN-γ in the culture supernatant were measured using a mouse IFN-γ ELISA kit (BD Biosciences), according to the manufacturer’s instructions. IFN-γ concentrations were compared as an index of the rate of CD8α^+^ lymphocyte proliferation.

### Comparison of the antigen-presenting capacity of CD11b^+^ spleen cells versus BM-DCs

To evaluate the capacity of antigen presentation by CD11b^+^ spleen cells and BM-DCs, either CD11b^+^ spleen cells (0.5×10^5^) or BM-DCs (0.5×10^5^) were co-cultured with doxorubicin-treated neuro-2a cells (2.0×10^5^) and CFSE-labeled CD8α^+^ cells (2.0×10^5^) in 24-well plates coated with hamster anti-mouse CD3/CD28 antibodies without CpG-ODN. Following three days of co-culture, CD8α^+^ cell proliferation was confirmed using FACS. Concentrations of IFN-γ in the culture supernatant were measured using a mouse IFN-γ ELISA kit (BD Biosciences).

### Evaluation of the ability of doxorubicin treatment to induce immunogenic cell death in neuroblastoma cells

To show that doxorubicin has the ability to induce immunogenic cell death in neuroblastoma cells, doxorubicin or CDDP-treated neuro-2a cells were co-cultured with BM-DCs and CD8α^+^ lymphocytes, and IFN-γ concentrations in the culture supernatants were compared. CDDP was used as a control agent as it is unable to induce immunogenic cell death in tumor cells (14). Neuro-2a cells (2.0×10^5^) treated with either doxorubicin (5 μM for 24 h) or CDDP (25 μg/ml for 72 h) were co-cultured with CFSE-labeled CD8α^+^ cells (2.0×10^5^) in 24-well plates coated with hamster anti-mouse CD3/CD28 antibodies (BD Pharmingen). Following three days of co-culture, CD8α^+^ cell proliferation was confirmed using FACS, and IFN-γ concentrations in the culture supernatants were measured using a mouse IFN-γ ELISA kit (BD Biosciences).

## Results

### Induction of neuro-2a cell death by doxorubicin and CDDP

**Figure 1:**
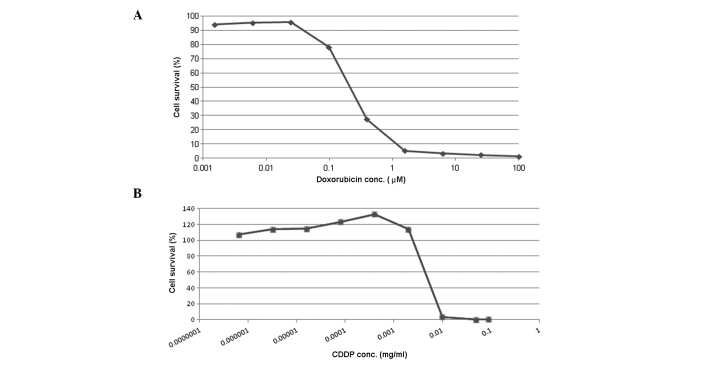
Doxorubicin or CDDP-induced cell death of neuroblastoma cells. (A) Neuro-2a cells were incubated with doxorubicin for 24 h, and the ratio of cell survival was measured. Incubation of neuro-2a cells with doxorubicin at concentrations >1.6 μM induced death in almost all cells. (B) When neuro-2a cells were incubated for 72 h with CDDP at concentrations >0.01 mg/ml, cell death was induced in almost all cells. CDDP, cisplatin.

The effect of doxorubicin and CDDP treatment on neuro-2a cell survival was dependent on the concentration of the two agents (Fig. 1). Following a 24-h incubation period, doxorubicin at concentrations of <0.1 μM caused minimal cell death, while at concentrations of >1.6 μM doxorubicin caused the majority of cells (>98%) to die (Fig. 1A). CDDP also induced neuro-2a cell death. Following a 72-h incubation period, >0.01 mg/ml CDDP caused death in >98% of cells (Fig. 1B).

### CD8α^+^ lymphocyte proliferation and IFN-γ production in co-culture with doxorubicin-treated neuro-2a cells and CD11b^+^ spleen cells

**Figure 2:**
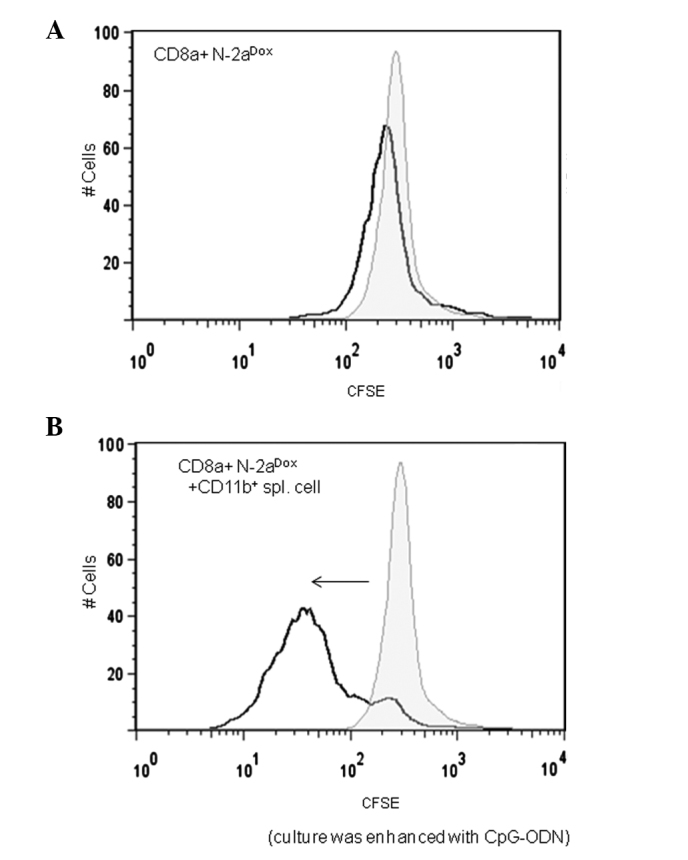
CD8α^+^ cell proliferation is induced by co-culture with CD11b^+^ spleen cells and doxorubicin-treated, dead neuro-2a cells. CD8α^+^ cells from mouse lymph nodes and spleens were stained with CFSE and co-cultured with CD11b^+^ spleen cells and doxorubicin-treated neuro-2a cells. CD8α^+^ cells were stimulated by anti-mouse CD3 and CD28 antibodies. After three days, CFSE dilution was analyzed by fluorescence-activated cell sorting. The gray shading represents the culture wells containing CD8α^+^ cells stimulated by CD3/28 antibodies. (A) CFSE dilution was not observed when CD8α^+^ cells were cultured only with CD11b^+^ spleen cells. (B) CFSE dilution was observed when CD8α^+^ cells were cultured with CD11b^+^ spleen cells and doxorubicin-treated neuro-2a cells. CD, cluster of differentiation; CFSE, carboxyfluorescein succinimidyl ester; CpG-ODN, CpG oligonucleotide.

When CD8α^+^ cells were cultured with neuro-2a cells alone, CD8α^+^ cell proliferation was not observed (Fig. 2A). However, when CD8α^+^ lymphocytes were co-cultured with CD11b^+^ spleen cells and doxorubicin-treated neuro-2a cells, FACS examination on the third day of the co-culture revealed a dilution of the CFSE. This was indicated by a reduction in the CFSE intensity of the CFSE-positive cell population relative to the initial staining. These data indicate that CFSE-labeled CD8α^+^ lymphocytes had proliferated and that the CFSE in the cytoplasm was diluted (Fig. 2B). CpG-ODN was added to the culture as an adjuvant for the lymphoproliferation reaction.

### Production of IFN-γ following co-culture of CD8α^+^ responder cells, CD11b^+^ APCs and doxorubicin-treated tumor cells under CD3/CD28 stimulation

**Figure 3:**
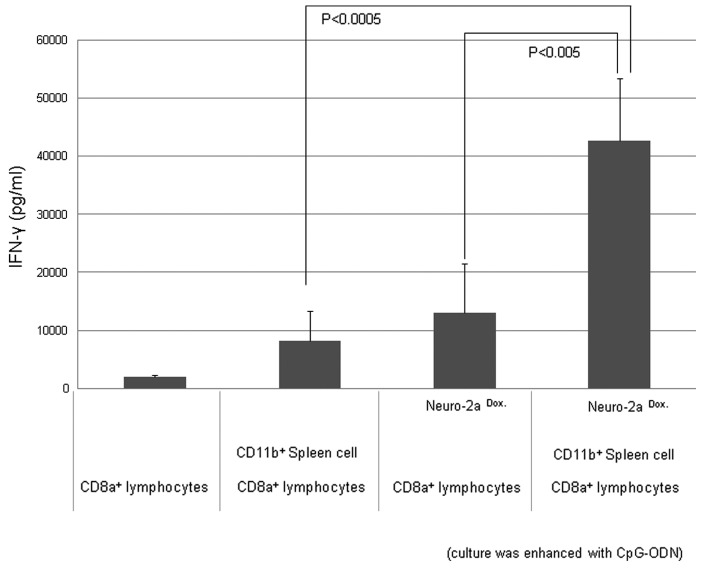
The concentration of IFN-γ in co-culture supernatants was measured using ELISA. IFN-γ production was markedly increased when CD8a^+^ cells were co-cultured with CD11b^+^ spleen cells and doxorubicin-treated neuro-2a cells accompanied by CD3/28 stimulation and CpG-ODN enhancement. CD, cluster of differentiation. IFN-γ, interferon-γ; CpG-ODN, CpG oligonucleotide.

IFN-γ production was observed when CD8α^+^ lymphocytes were co-cultured with CD11b^+^ spleen cells and doxorubicin-treated neuro-2a cells with stimulation by CD3 and CD28 antibodies, and CpG-ODN as an adjuvant (Fig. 3).

### BM-DCs induce IFN-γ production more effectively than CD11b^+^ spleen cells

**Figure 4:**
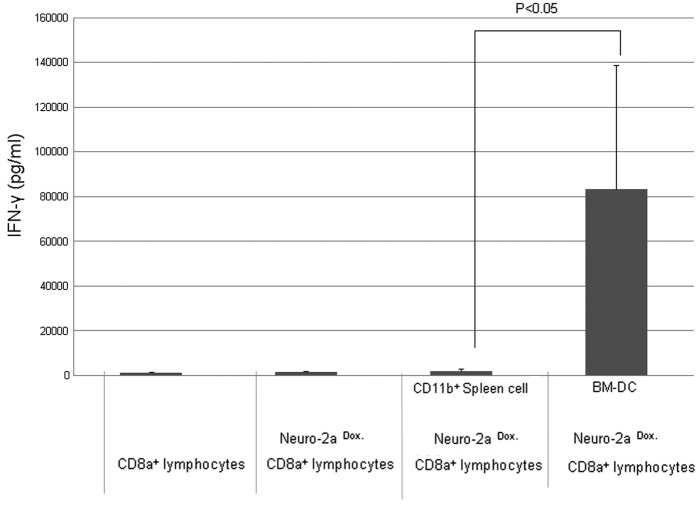
Comparison of the antigen-presenting capacity of CD11b^+^ spleen cells and BM-DCs. CD8a^+^ cells were co-cultured with either CD11b^+^ spleen cells or BM-DCs, accompanied by doxorubicin-treated neuro-2a cells and CD3/28 stimulation without CpG oligonucleotide enhancement. Higher levels of IFN-γ production were observed in supernatants from cultures containing BM-DCs compared with those containing CD11b^+^ spleen cells. CD, cluster of differentiation. IFN-γ, interferon-γ; BM-DC, bone marrow-derived dendritic cell.

When CD8α^+^ lymphocytes were cultured using BM-DCs as the APCs, IFN-γ production was markedly increased compared with cultures using CD11b^+^ spleen cells. Enhancement by CpG-ODN was not necessary to confirm the promotion of IFN-γ production in culture using BM-DCs (Fig. 4)

### Doxorubicin effectively induces immunogenic cell death of neuroblastoma cells compared with CDDP

**Figure 5:**
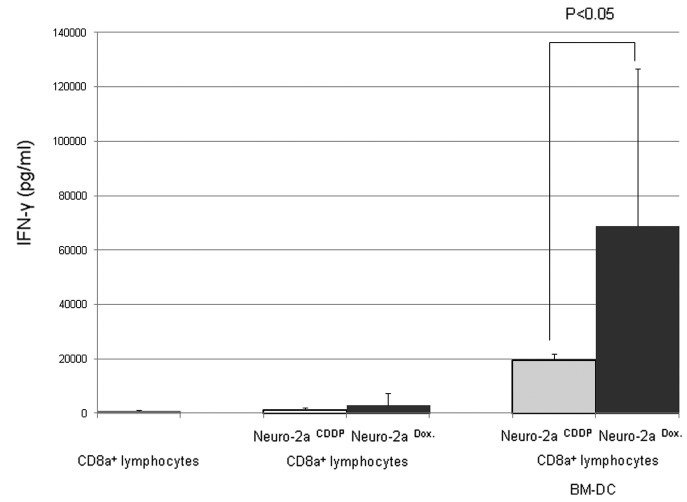
Doxorubicin induces immunogenic cell death in mouse neuroblastoma cells. The concentration of IFN-γ in the supernatants from co-cultures with doxorubicin-treated (black bar) or CDDP-treated (gray bar) neuro-2a cells was compared. When doxorubicin-treated neuro-2a cells were used, the concentration of IFN-γ in the supernatant was higher than when CDDP-treated neuro-2a cells were used. A stronger lymphoproliferative reaction was induced when neuroblastoma cells were treated with doxorubicin relative to CDDP. CD, cluster of differentiation. IFN-γ, interferon-γ; BM-DC, bone marrow-derived dendritic cell; CDDP, cisplatin.

As shown in Fig. 5, when doxorubicin-treated neuro-2a cells were co-cultured with BM-DCs and CD8α^+^ lymphocytes, IFN-γ production was increased compared with that when CDDP-treated neuro-2a cells were used. Thus, doxorubicin was shown to be able to induce immunogenic cell death in neuroblastoma cells. CDDP was used as a control agent as it is unable to induce immunogenic cell death in tumor cells (14). These results highlight the potential advantages of doxorubicin as it not only causes tumor cell death, but also induces antitumor lymphocyte reactions against the neuroblastoma cells.

## Discussion

Intensive multi-agent chemotherapy is the key to the induction and consolidation of remission in advanced neuroblastoma. However, cell-based immunotherapy is another promising approach. With the aim of improving the prognosis of patients with advanced neuroblastoma, this study analyzed the interaction between these two therapeutic strategies. It was hypothesized that immunogenic cell death and the subsequent innate cellular immune reactions are particularly important.

Engulfment of apoptotic tumor cells by innate immune cells, such as macrophages or DCs, induces sequential immune reactions. These innate cellular phagocytes are considered to have a pivotal role in the mechanism of tumor immunity (2,14–17). In particular, BM-derived immature DCs phagocytose, mature and later contribute to immune reactions against cancer cells (2,13,18,19). Alternatively, tumor-associated macrophages infiltrate the tumor, engulfing dead cells and inducing a tolerogenic reaction to tumor cells (15,16,20). Moreover, the induction of apoptotic tumor cell death by chemotherapeutic agents and the subsequent immunogenic reactions are important factors in the immunological antitumor reaction. Using murine colon cancer (CT26) and fibrosarcoma (MCA205) models, Obeid *et al* (5) reported that anthracyclins induce immunogenic tumor cell death via CRT exposure on the cell surface. In these models, it has been suggested that dying cancer cells with exposed CRT are phagocytosed by DCs, prior to a sequential antitumor immune reaction being induced (21).

Although controversial, a previous study reported that circulating tumor cells (CTCs) contribute to the development of metastases in neuroblastoma (22). It was proposed that CTCs, which are undetectable using conventional radiological and microscopic techniques, spread systematically and are regulated by a different molecular mechanism to that of the primary tumor lesion (23). Therefore, in the present study, it was hypothesized that the doxorubicin-induced immunogenic cell death of neuro-2a cells was likely to induce an immune reaction following phagocytosis by innate cellular phagocytes. To confirm the mechanism of action, several co-culture experiments were performed. When CD8α^+^ lymphocytes were stimulated by CD3/CD28 antibodies and co-cultured with doxorubicin-treated neuro-2a cells and CD11b^+^ cells, a marked increase in the proliferation of CD8α^+^ lymphocytes and IFN-γ production was observed. These results indicate that CD11b^+^ cells phagocytose dead tumor cells and induce activation of CD8α^+^ cytotoxic T-lymphocyte proliferation. Moreover, phagocytosis of apoptotic cells by BM-DCs markedly increased IFN-γ production. This mechanism may have contributed to the antitumor effect of doxorubicin on mouse neuroblastoma cells.

Data from the present study indicate that doxorubicin is capable of inducing immunogenic neuroblastoma cell death. Previously, chemotherapy agents have been regarded as detrimental from an immunological point of view due to their myelosuppressive effect. However, recent studies have suggested that chemotherapy may increase tumor immunity (4,24). Induction of immunogenic cell death represents a key mechanism for augmentation of tumor immunity. Results from the present *ex vivo* study indicate that induction of neuroblastoma cell death by doxorubicin may enhance survival of tumor-bearing mice *in vivo* and promote proliferation of CD8α^+^ lymphocytes and IFN-γ production *in vitro*. Since cell death of neuro-2a cells was induced *ex vivo*, the immunogenic effect of doxorubicin occurs independently of the host immune system. This demonstrates that doxorubicin has an advantageous immunological antitumor effect on neuroblastoma cells via the induction of immunogenic cell death. In this mechanism, phagocytosis by BM-DCs is considered to contribute to the rejection of neuro-2a cells.

In children with advanced neuroblastoma, initial induction of remission by intensifying chemoradiotherapy improves the survival rate. However, a number of children exhibit relapses following initial treatment, which is likely due to neuroblastoma cells acquiring resistance to the antitumor mechanism (10–12). Novel insights into the effects of conventional therapy may improve current therapeutic regimens, and the development of innovative therapies for advanced neuroblastoma remains particularly important. Analysis of the immunological effect of conventional chemotherapeutic agents and clinical trials of antitumor immunological therapies are likely to contribute to further increase the survival rate of patients with advanced neuroblastoma. In conclusion, the investigation of the immunological efficacy of conventional antitumor agents provides useful information for improving intensive chemotherapy and for the development of immunological approaches for neuroblastoma therapy.
